# Evaluating the impact of Relative Total Dose Intensity (RTDI) on patients' short and long-term outcome in taxane- and anthracycline-based chemotherapy of metastatic breast cancer- a pooled analysis

**DOI:** 10.1186/1471-2407-11-131

**Published:** 2011-04-12

**Authors:** Sibylle Loibl, Tomas Skacel, Valentina Nekljudova, Hans Joachim Lück, Matthias Schwenkglenks, Thomas Brodowicz, Christoph Zielinski, Gunter von Minckwitz

**Affiliations:** 1 German Breast Group, Neu-Isenburg, Germany; 2 First Faculty of Medicine, Clinical Department of Haematology, Charles University Prague, Czech Republic; 3 Amgen Europe, Zug, Switzerland; 4 Gynaecologic Oncology Clinic, Hannover, Germany; 5 European Center of Pharmaceutical Medicine, University of Basel, Switzerland; 6 Clinical Division of Oncology, General Hospital and Central European Cooperative Oncology Group (CECOG), Medical University, Vienna, Austria

## Abstract

**Background:**

Chemotherapy dose delay and/or reduction lower relative total dose intensity (RTDI) and may affect short- and long-term outcome of metastatic breast cancer (MBC) patients.

**Methods:**

Based on 933 individual patients' data of from 3 randomized MBC trials using an anthracycline and taxane we examined the impact of RTDI on efficacy and determined the lowest optimal RTDI for MBC patients.

**Results:**

Median time to disease progression (TTDP) and overall survival (OS) of all patients were 39 and 98 weeks. Overall higher RTDI was correlated with a shorter TTDP (log-rank p = 0.0525 for 85% RTDI cut-off). Proportional hazards assumption was violated, there was an early drop in the TTDP-curve for the high RTDI group. It was explained by the fact that patients with primary disease progression (PDP) do have a high RTDI per definition. Excluding those 114 patients with PDP the negative correlation between RTDI and TTDP vanished. However, non-PDP patients with RTDI-cut-off levels <85% showed a shorter OS than patients with higher RTDI levels (p = 0.0086).

**Conclusions:**

Optimizing RTDI above 85% appears to improve long-term outcome of MBC patients receiving first-line chemotherapy. Lowering RTDI had no negative influence on short term outcome like OR and TTDP.

## Background

Therapy of metastatic breast cancer (MBC) has improved over the last decades probably due to sequential systemic treatment in multiple lines[1]. Anthracyline and taxane-based therapies are part of the standard of care in first line MBC. More than 50% of patients receive sub-optimally dosed adjuvant treatment. Evidence-based recommendations for chemotherapy dosing in the metastatic setting are expected to be followed even less stringently, it is not known whether this results in a detrimental course of the disease and earlier death[2]. Over the past 40 years clinical trials have sought to determine benefit of greater chemotherapy dose intensity. The consequences of dose delays and reductions are significant, potentially affecting short and long-term outcomes as well as patient's QoL. Maintaining the dose and schedule are of central concern in delivery of chemotherapy. The Goldie-Coldman hypothesis predicts that delivering higher doses of chemotherapy agents reduces the survival probability of chemotherapy-resistant clones[3,4]. The Norton-Simon hypothesis advances previous theories by incorporating the concept of chemotherapy schedule[5]. These hypotheses highlight the role of dose and schedule and have significantly influenced oncology practice. The intended dose is usually reported as part of the design of clinical studies, but a review of large randomized chemotherapy trials conducted between 1990 and 2000 found that the relative total dose intensity (RTDI) is poorly reported in the literature [6]. Bonadonna et al [7] as well as the surveys of community practice patterns show that a substantial proportion of patients with cancer are given less than 85% of the suggested dose[8]. A retrospective analysis of community practice data from records of 20,799 patients with early breast cancer (EBC) between 1997-2000 found that the average actual RTDI was 79% [2]. In this analysis, 56% of patients with EBC received less than 85% RTDI. Significant dose reductions and treatment delays are common clinical practice in the management of patients with primary breast cancer. Even more alarming picture might be observed in patients with MBC, where dose delays and reductions are common methods of reducing the toxicities and maintain the QoL.

Therefore, in this analysis the impact of relative total dose intensity in first line metastatic breast cancer patients treated with anthracycline/taxane based therapy has been investigated.

## Methods

### Study identification

We conducted a Medline literature search (Feb 2006) to identify prospective randomized phase 3 clinical trials which evaluated efficacy and safety of anthracycline/taxane-based chemotherapy regimens in patients with MBC and were published between 1995-2006. A supplementary search covered abstracts from the ASCO annual meetings (1995-2006). The search produced 51 relevant articles. Then, further limitations were added, i.e. completed MBC studies were eligible if: (i) they were phase 3 prospective interventional clinical trials performed in accordance with GCP, (ii) they evaluated short (ORR) and long-term (TTDP, OS) efficacy measures, clearly specified dosing of chemotherapy, including guidance for dose delays and reductions, collected toxicity data, and had a minimum follow-up period of 24 months.

A total of 7 eligible studies were identified from the original literature search. The key consideration for final inclusion into the analysis was timely access to individual patient data (by March 2006) to allow for a prospective planning of this analysis. Accordingly, three randomized clinical trials were included into this integrated analysis.

1. CECOG: n = 258; phase 3 study of gemcitabine (1000 mg/m^2 ^i.v. d1,4), epirubicin (90 mg/m^2 ^d1) and paclitaxel (175 mg/m^2 ^d1), GET Q3W for a maximum of 8 cycles vs. FU (500 mg/m^2 ^d1), epirubicin (90 mg/m^2 ^d1), and cyclophosphamide (500 mg/m^2 ^d1), FEC Q3W for a maximum of 8 cycles as first-line chemotherapy in MBC[9].

2. AGO Mamma-1**: **n = 514; phase 3 study of epirubicin (60 mg/m^2 ^d1) and paclitaxel (175 mg/m^2 ^d1), ET Q3W compared with epirubicin (60 mg/m^2 ^d1) and cyclophosphamide (600 mg/m^2 ^d1), EC Q3W for a maximum of 10 cycles as a first-line chemotherapy in MBC[10].

3. AGO Mamma-3**: **n = 164 of 340 randomized; phase 3 study of epirubicin (60 mg/m^2 ^d1) and paclitaxel (175 mg/m^2 ^d1), ET Q3W compared with paclitaxel (175 mg/m^2 ^d1) and capecitabine (2000 mg/m^2 ^days 1-14), TX Q3W for a maximum of 6 cycles as a first-line chemotherapy in MBC[11].

The TX arm of the Mamma-3 study was not included into the pooled analysis, since the administration of capecitabine p.o. Q2W did not match the inclusion criteria.

All trials were performed according to the Declaration of Helsinki.

### Study population

This statistical analysis was based on individual subject data of 934 patients obtained from the above specified phase 3 MBC trials.

### Objectives and outcome measures

The objectives of this study were to determine impact of reduced Relative Total Dose Intensity (RTDI) on short-term (Overall Response Rate, ORR) and long-term (Time-to-Disease Progression, TTDP and Overall Survival, OS) outcome measures, to identify potential predictors for TTDP and OS and determine optimal RTDI for MBC patients treated with the anthracycline and taxane-based first-line treatment.

### Indicators of chemotherapy delivery

Relative Total Dose Intensity (RTDI) is the ratio of Actual Total Dose Intensity (ATDI) and Planned Total Dose Intensity (PTDI), expressed as a percentage.

Planned total dose intensity (PTDI) is the planned dose intensity over the entire treatment duration, averaged across the chemotherapy agents used. In case of permanent treatment discontinuation, other than disease progression (DP) or death, the remaining cycles are calculated with the planned length and zero dose. For patients who withdrew from chemotherapy due to DP or death the PTDI was calculated based on the number of cycles actually completed.

Actual total dose intensity (ATDI) is defined as the actual average dose intensity over the real treatment duration

Note that RTDI expresses the effect of reductions, delays as well as premature discontinuations in a treatment (due to the reasons other than disease progression or death).

The maximal body surface area (BSA) for calculation of dosage was restricted in the CECOG study at 2.2 m^2^, and in the Mamma-3 study at 2.0 m^2^. The BSA was not limited in the Mamma-1 study. RTDI was first calculated separately for each component of the chemotherapy regimen (e.g. anthracycline). Then for each combination chemotherapy regimen (e.g. EC) an average across the components was taken to derive the RTDI of the combination.

TTDP is defined as the time in weeks between the date of first chemotherapy application and the date of the first documented sign of disease progression or death due to any cause. OS is defined as the time in weeks between the date of first chemotherapy application and death due to any cause. Patients who withdrew consent or were lost to follow-up were censored at the moment of the last contact.

Primary Disease Progression (PDP) is defined as the disease progression or death which occurred before or at the first primary disease status evaluation (i.e. within first 2 (CECOG) or 3 (AGO-Mamma-1 and Mamma-3) cycles).

### Integrated analysis and statistical evaluation

We carried out an integrated analysis of the individual patient data. Descriptive summaries of demographic and disease characteristics for included patients were prepared by study and for the integrated populations within each treatment group.

Data were analyzed with SAS^® ^Version 9.2 for Windows under SAS Enterprise Guide^® ^Version 4.1 (SAS Institute Inc. Cary NC, USA).

Categorical parameters were summarized by the number and percentage of patients in each category. Continuous parameters were summarized by mean, median, minimum and maximum values. Two-sided 95% confidence intervals were presented, where appropriate. All statistical tests were two-sided. The default significance level was 5%. For all multivariate regression models (binary logistic, Cox) the models were selected with the backward elimination with sls (significance level to stay) = 0.2 starting with all potential predictors listed for the model. RTDI was forced into the Cox and logistic regression models because it is a key factor of interest. Proportionality of hazards was assessed for the Cox models with log-minus-log plots.

The following baseline covariates were assessed for possible significant effects on the endpoints: age (years), ECOG PS, ER/PgR status, histological tumor type (ductal vs. lobular vs. other), nodal status at primary diagnosis (N0 vs N+), tumor size at primary diagnosis (T1-3 vs. T4), previous chemotherapy (none vs. non-anthracycline vs. anthracycline containing), previous endocrine therapy, metastatic site (low (bone, skin, lymphatic nodes, lung only) vs. high risk (liver, CNS, lung+other)) and chemotherapy arm. The following planned predictors were not considered as covariates since they were not available for all three studies; HER/2/neu, tumor grading and previous radiotherapy.

At first, univariate Cox-proportional hazards models for TTDP and OS with continuous RTDI as a single predictor were fit. A squared term for RTDI was tested in order to account for potential non-linearity of relationship between RTDI and outcome. However, these models fit poorly according to the supremum test for the functional form [12]. The addition of the squared term did not improve the situation, other possible transformations of continuous predictor also seemed inappropriate. Therefore, all following Cox proportional hazard's models for TTDP and OS, both univariate and multivariate anylsis, were finally fit for different binary versions of RTDI for pre-specified cut-points (95%, 90%, 85%, 80% and 75%). The Kaplan-Meier estimators of survival function were computed for each RTDI group, the corresponding curves were presented graphically and the log-rank test was performed to compare the time-to-event outcome across RTDI groups[13].

## Results

### Baseline

934 patients were included in the final analysis according to the inclusion criteria. 114 (12%) patients experienced a Primary Disease Progression (PDP). One patient had an implausible date of progression and was excluded from all analyses. The median age of the study population was 56 years. Baseline characteristics are outlined in Table 1.

**Table 1:** Patient characteristics

	Study						Overall N = 934	
			
	CECOG GET vs FEC N = 256		AGO Mamma 1 N = 514		AGO Mamma 3 N = 164			
**Age, years**								

**median (range)**	53.5 (29-74)		56.0 (28-75)		58.0 (21-76)		56.0 (21-76)	

	**N**	**%**	**N**	**%**	**N**	**%**	**N**	**%**

**HR status**								
								
**ER and/or PgR positive**	118	67.43	324	69.83	131	80.37	573	71.45

**missing**	81		50		1		132	

**Her2**								
								
**positive**	-	-	-	-	30	23.08	30	23.08

**missing**	256		514		34		804	

**Nodal status at first diagnosis**								
								
**positive**	164	65.08	282	65.58	95	67.86	541	65.82

**missing**	4		84		24		112	

**ECOG PS**								
								
**0**	122	47.66	237	46.11	104	65.00	463	49.78

**1 or 2**	134	52.34	277	53.89	56	35.00	467	50.22

**missing**	0		0		4		4	

**Prior chemotherapy**								
								
**none**	126	49.22	325	63.98	96	58.54	547	58.94

**non-anthracycline**	130	50.78	162	31.89	34	20.73	326	35.13

**anthracycline**	0	0	21	4.13	34	20.73	55	5.93

**missing**	0		6		0		6	

**Prior endocrine therapy**								
								
**yes**	99	38.67	220	44.62	85	51.83	404	44.25

**missing**	0		21		0		21	

**Metastatic site**								
								
high-risk*	207	80.86	318	64.50	113	68.90	638	69.88

**missing**	0		21		0		21	

The median follow-up was 72 weeks for the whole study population (67.0w in CECOG, 75.0w Mamma-1, 71.5w Mamma-3, respectively).

Overall 27.3% of the patients discontinued due to progression or death and 18.1% due to other, non specified reasons. The rate of discontinuation was lowest (18.3%) in the Mamma-3 trial (planned 6 cycles) and approximately 50% in the two other trials (planned up to 8 and 10 cycles). Dose reductions were mainly due to toxicity (though it is unknown whether it was exactly according to the protocol). The majority of dose delays (from 30% to 50% for individual studies) were due to logistic reasons or patients wish.

### Primary outcome measure

The median RTDI for the whole study population was 95.5% (CECOG 88.8%, Mamma-1 95.6%, Mamma-3 99.6%), whereas the mean RTDI overall was 85.7% (CECOG 81.2%, Mamma-1 85.6%, Mamma-3 93.0%).

**Figure 1:**
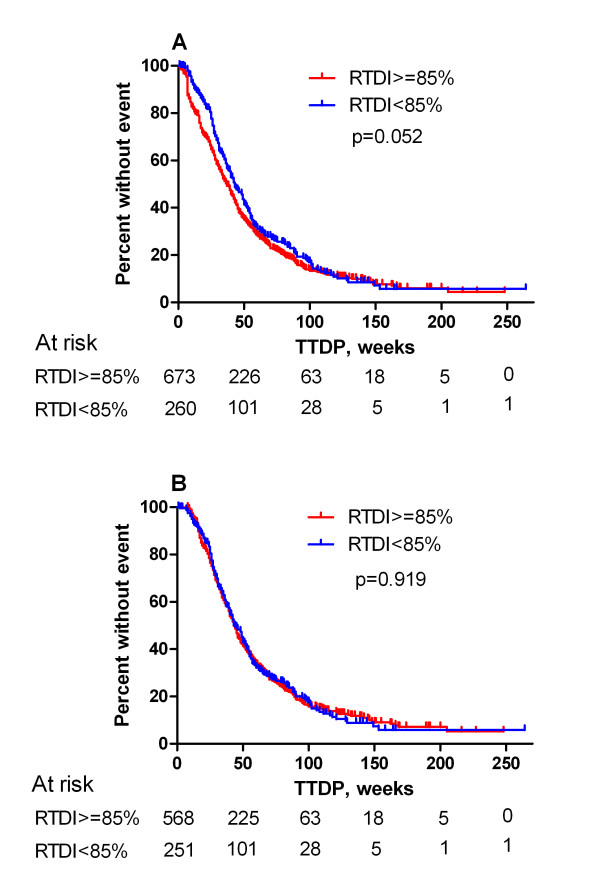
**Kaplan-Meier curve for TTDP**. A: Kaplan-Meier for TTDP; 85% RTDI level (all pts). The early drop in the curve for patients with high RTDI is clearly shown. B: Kaplan Meier curve for TTDP; 85% RTDI level without patients with PDP.

**Figure 2:**
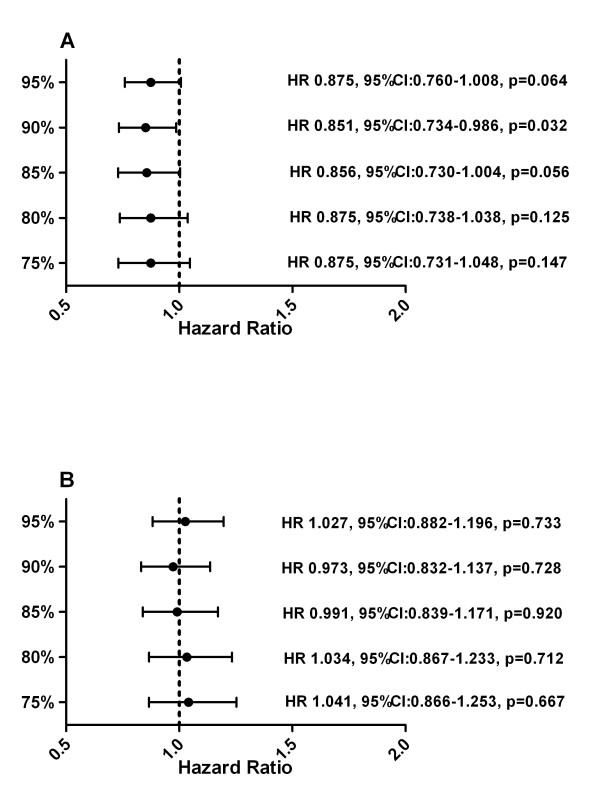
**Hazard Ratio for Time To Disease Progression (A) with and (B) without patients with PDP**.

Results of all 933 eligible patients showed that high RTDI had a negative impact (p > 0.05 for all cuts except 90%) on TTDP in the univariate analysis. Patients with higher RTDI had a shorter TTDP compared to those with lower RTDI (85% RTDI level; 37 weeks vs. 43 weeks (p = 0.0525); Figure 1). Proportionality of hazards was violated. Adding a time-dependent term also did not model the data adequately, since this non-proportionality was caused by a short early drop in the Kaplan-Meier curve for TTDP in the low RTDI group. However, the analysis of TTDP excluding 114 (12%) patients with the PDP who have high RTDI by definition showed no difference between high and low RTDI group (Figure 1 and 2). The backward selection multivariate analysis for the different pre-specified RTDI cut-off levels excluding the PDP revealed only the following factors as significant prognostic factors: HR status, ECOG, metastatic site and previous chemotherapy.

### Secondary outcome measures

**Figure 3:**
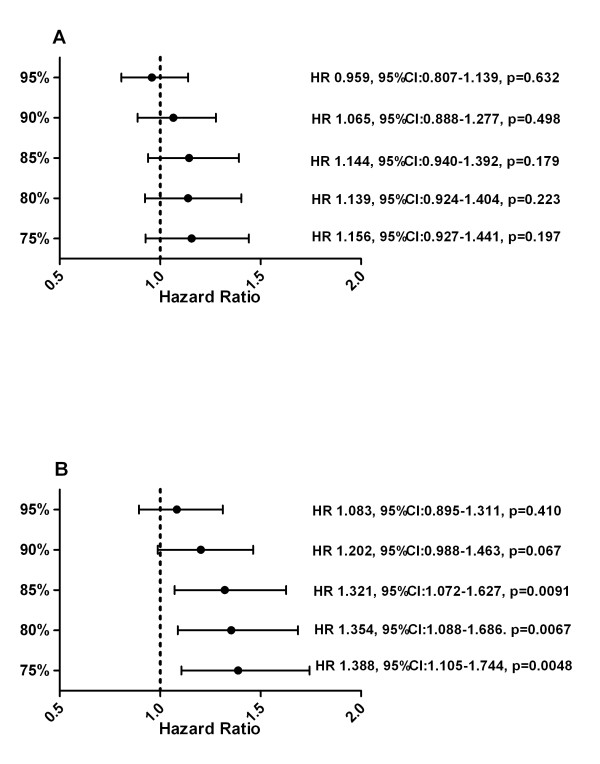
Hazard Ratio for OS (A) with and (B) without patients with PDP.

**Figure 4:**
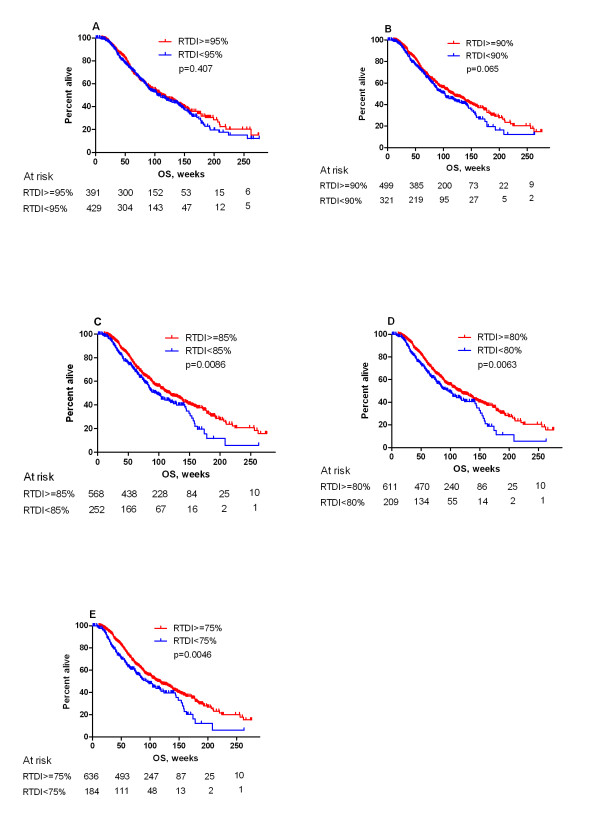
**Kaplan-Meier for overall survival excluding patients with Primary Disease Progression (n = 820) according to RTDI binary cuts**. A: 95% (median OS: 112 weeks (95% CI [97-136]) for RTDI ≥ 95% vs. 104 weeks (95% CI [91-129]) for RTDI < 95%. B: 90% (The median OS was 118 weeks (95% CI [103-138]) for RTDI ≥ 90% vs. 103 weeks (95% CI [86-120]) for RTDI < 90%.); C: 85% (The median OS was 118 weeks (95% CI [103-136]) for RTDI ≥ 85% vs. 96 weeks (95% CI [83-119]) for RTDI < 85%). D: 80% (The median OS was 116 weeks (95% CI [103-133]) for RTDI ≥ 80% vs. 96 weeks (95% CI [77-124]) for RTDI < 80%.) E: 75% (The median OS was 113 weeks (95% CI [103-133]) for RTDI ≥ 75% vs. 93 weeks (95% CI [76-119]) for RTDI < 75%.

The median OS for the whole study population was 98 weeks. The OS for all subjects, including patients with PDP, showed a non significant trend in favor of patients with high RTDI (Figure 3a). When patients with the PDP (n = 114) were excluded from the analysis, the influence of RTDI on OS became significant. The Kaplan-Meier curve showed that patients with lower RTDI had shorter OS. (Figure 4a-e). The lower the cut-off level, the more prominent was the effect of low RTDI on the OS (RTDI<90% OS of 103 weeks, RTDI<85% OS of 96 weeks and RTDI<75% OS of 93 weeks).

The hazard ratios calculated by Cox model are given in Figure 3b. The multivariate analysis (n = 585) showed a non-significant trend towards improved OS for patients with high RTDI. Factors independently influencing OS were HR status, ECOG, metastatic site, previous chemotherapy, therapy arm (borderline significant).

**Figure 5:**
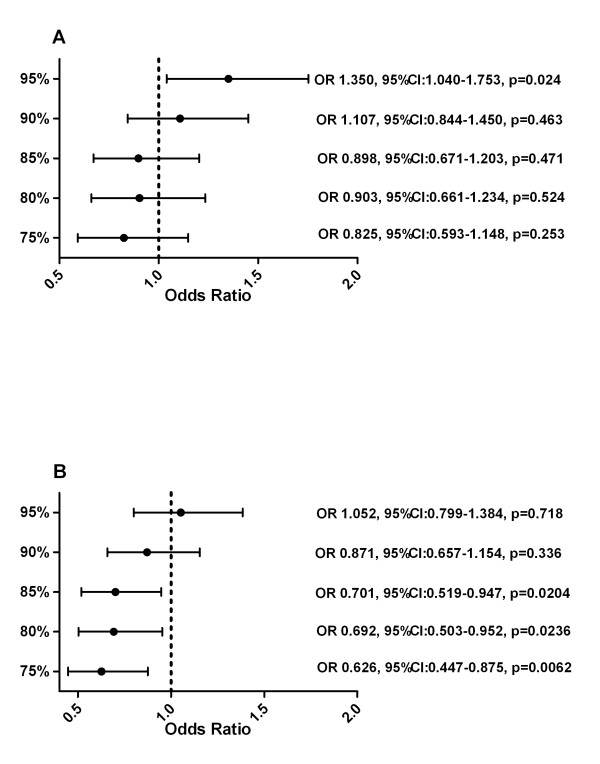
**Odds Ratio for ORR (A) with and (B) without patients with PDP**.

The full set analysis showed partly negative correlation between the RTDI and ORR for the 95% (p = 0.024) and 90% cut off. (Figure 5a) When patients with the PDP were excluded, positive correlation was seen for all cuts except 95%, which were significant for 85% cut and lower. (Figure 5b).

## Discussion

This pooled analysis based on individual patients' data from three randomized phase 3 clinical trials (recruited 1996-2005) was designed to address an important clinical question, whether dose-delays and/or dose reduction do impact long-term (TTDP, OS) and short-term (ORR) outcome for patients with MBC treated with first line anthracycline/taxanes-based therapy. To the best of our knowledge, the prognostic value of RTDI on long and short-term outcomes in MBC has not been systematically addressed, neither in a prospective nor retrospective fashion.

It is not possible to put our results into the context with other MBC studies, because no comparable analyses were reported in this setting. The only published data were generated in the adjuvant setting by Bonadonna et al. [7]. They demonstrated that patients who received 85% or more of the optimal dose of CMF had a longer relapse free and OS. Patients with less than 85% relative dose had a survival comparable to those without adjuvant chemotherapy. These data are first of all based on CMF as adjuvant treatment and second did not take into account the administration period. That dose matters in the adjuvant setting has been shown by several other groups (e.g. CALGB) and led to the dose-intensified and high dose chemotherapy approaches [14,15].

However, population involved and the aims of treatment in the advanced disease are different compared to the adjuvant setting and several reasons other than the physician's decision may intervene in determining the reduction/delay of chemotherapy treatment in patients who by definition are not curable and may also have some disease-related impairments which limit the administration of full dose therapy. Quality of life (QoL) is consistently considered a main objective in evaluating the impact of treatments in advanced disease. Treatment is aimed to ameliorate disease-related symptoms but it may also provoke toxicities impairing QoL. This has not been considered in this analysis.

Looking at our entire study population, we did observe that maintaining the planned first-line taxanes/anthracyclines-based therapy on time and schedule led to a decreased TTDP and OR. We identified this as an artefact driven by two main reasons: a significantly higher number of PDPs (114 pts, 12%) than initially anticipated, and our definition of RTDI for progressing patients. If a patient progressed within 2 or 3 cycles of treatment, the last dose/schedule was carried forward for remaining planned cycles and used for calculation of the RTDI. This led to an artificially high RTDI for 12% of the patients with early progression. After adjustment for PDP the median OS was significantly improved for patients with high RTDI (e.g. for RTDI ≥85% 118 weeks compared to 96 weeks in the group with RTDI <85%; p = 0.0086.

The TTDP was comparable for the patient populations with high as well as low RTDI. These OS results were not confirmed to be significant in the multivariate Cox model, probably due to the reduced patient numbers (n = 585) included in this analysis. The KM estimation for the patients included in the multivariate analysis showed an insignificant trend for improved OS with higher RTDI. However, an influence by other factors cannot completely be excluded. It is unusual that the OS but not the TTDP was impacted by the RTDI. This is in contrast to results of most recently published MBC studies, where improvement of TTDP did not result in improvement of the OS. It is well known that OS is never influenced by the initial therapy alone, and that subsequent multiple treatments, including second-line chemotherapy, radiotherapy, supportive care, diet and social environment play critical role. It might be that patients who receive optimal RTDI in the 1^st ^line setting will do so for the forthcoming therapy lines. This would support the hypothesis that maintaining RTDI in the first-line chemotherapy is also critical for OS of MBC.

Another objective of this study was to determine the optimal RTDI cut-off level for studied population. We found out that any reduction of RTDI level has negative effect on OS. In our study, the significant improvement of OS was observed starting from the RTDI of 85% (HR 1.32). This is probably due to the sample size in respective cut-off levels.

To determine which variables had independent prognostic value and to control for the difference between studies, we performed a multivariate analysis. In our series of patients, ECOG 1,2 (vs. 0), high-risk metastasis (vs. low-risk) and previous chemotherapy were associated with significantly worse prognosis both for TTDP and OS. Positive HR status was associated with improved TTDP and OS. The strengths of our study are: large sample size, the analysis is based on 934 individual patient's data; all three phase 3 trials investigated the role of current standard treatment (taxane/anthracycline-based chemotherapy); and the majority (73%) of the data were generated in one country only, therefore it is probable that second-line treatment algorithm and supportive care management were similar for those 680 patients. The analysis also has some limitations. Its retrospective nature which was addressed by prospective planning of the analysis. One of the study (AGO-Mamma-1) had a larger sample size as compared to two other ones. Paclitaxel/capecitabine arm was excluded from the analysis due to inaccurate dose/time reporting of capecitabine. No data on further treatment were available. Quality of life data were not evaluated in this analysis. Lastly, most of the patients (680 pts) received lower than currently recommended dose of epirubicin (60 mg/m^2 ^in AGO-Mamma 1 and 3) which might have impacted the outcome for those patients.

## Conclusions

Optimizing RTDI above 85% for patients with MBC appears therefore a reasonable goal seems to be important to improve long-term outcome of MBC patients receiving first-line anthracycline/taxane-based chemotherapy. Lowering RTDI had no negative influence in the short run, but OS was better in patients with high RTDI. Dose reductions and delays should only be undertaken due to important reasons as unacceptable toxicity impairing quality of life.

## List of abbreviations

RTDI: relative total dose intensity; MBC: metastatic breast cancer; QoL: quality of life; TTDP: median time to disease progression; OS: overall survival; BSA: body surface area; PDP: primary disease progression; Sls: significancese level to stay; ORR: overall response rate; PDP: primary disease progression; CECOG: Central European Cooperative Oncology Group; AGO: Arbeitsgemeinschaft Gynäkologische Onkologie; DP: disease progression; PTDI: planned total dose intensity; ATDI: actual dose intensity.

## Competing interests

The authors declare that they have no competing interests.

## Authors' contributions

SL, TS and VN carried out the statistical analysis. SL, TS,VN drafted the manuscript, SL, TS, VN, MS, HJL, TB, CZ, GvM participated in the design and coordination of the study,. All authors read and approved the final manuscript.

## Pre-publication history

The pre-publication history for this paper can be accessed here:

http://www.biomedcentral.com/1471-2407/11/131/prepub
